# Primary Pituitary Tubercular Abscess: A Case Report

**DOI:** 10.31729/jnma.4433

**Published:** 2019-06-30

**Authors:** Yam Bahadur Roka, Narayani Roka, Sagar Raj Pandey

**Affiliations:** 1 Department of Neurosurgery, Neuro Cardio and MultiSpeciality Hospital, Biratnagar, Nepal; 2 Department of Ophthalmology, Neuro Cardio and MultiSpeciality Hospital, Biratnagar, Nepal; 3 Department of Pathology, Neuro Cardio and MultiSpeciality Hospital, Biratnagar, Nepal

**Keywords:** *abscess*, *pituitary gland*, *pyogenic*, *sella*, *tuberculosis*

## Abstract

Primary pituitary tubercular abscess is a very rare disease. It may present clinically with visual loss, headache, seizure, hormonal abnormalities or with cranial nerve palsies. MRI is the diagnostic modality and shows a cystic-solid mass in the sellar and suprasellar region, isointense on T1 and T2W images with heterogeneous areas and ring enhancement on contrast. Surgery remains the initial treatment and it is approached through the trans-sphenoidal/trans-nasal or transcranial route followed by anti-tubercular therapy. We report a case of primary pituitary tubercular abscess managed successfully with a brief review of its pathology.

## INTRODUCTION

Primary pituitary tubercular abscess (PTA) is a very rare disease.^1–4^ Tuberculoma of the sellar region and intracerebral tubercular abscess are more common than tubercular pituitary abscess. Unlike pyogenic abscess which can spread by both hematogenous or local spread, PTA is entirely secondary to hematogenous spread. It may present clinically with visual loss, headache, seizure, hormonal abnormalities or with cranial nerve palsies.^5^ Radiologically, it can mimic a pituitary adenoma, arachnoid cyst, colloid cyst, pyogenic abscess, metastasis or craniopharyngioma. The majority are managed with an initial diagnosis of adenoma with postoperative histopathology showing PTA.

## CASE REPORT

A 34- year old female presented in January 2014 with complaints of progressive loss of vision and headache for the past 3 months. For her decreased vision, she was prescribed glasses and analgesics but there was no relief of her symptoms. There was no other significant medical history, she was married with two children and normal menstrual cycle.

On general examination she was conscious, oriented and vitals were stable. On ophthalmological examination her visual acuity on the right eye was 6/60 and left eye was 4/60 with slight improvement on her vision with glasses. There was ptosis and dilatation of pupil on the right eye suggestive of third nerve palsy. Examination of pupil on the left eye was normal and reactive. Further examination of fundus showed temporal pallor of both discs. Humphreys visual field (HVF) showed bitemporal hemianopia.

**Figure 1.**
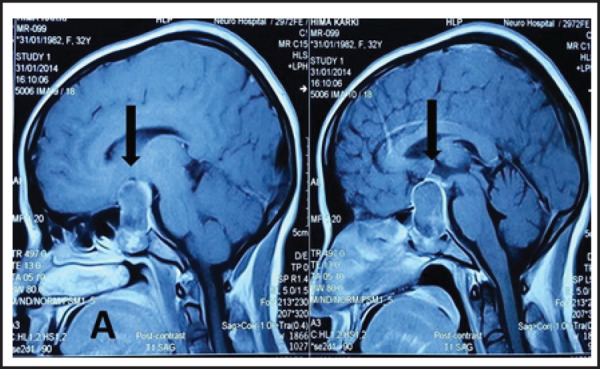
MRI showing a large cystic lesion in the sellar area with rim enhancement mimicking a pituitary adenoma.

Investigations showed abnormal thyroid function test (TFT) [TSH>40 m UI (normal 0.5 to 5)] with other hormones within normal limits. Magnetic resonance imaging (MRI) showed a large sellar mass with primarily suprasellar extension involving both the optic nerves and inter-carotid space (Figure 1). The lesion was extending lateral to the right carotid artery. T2 weighted image showed central hyper intense signal and there was thick ring enhancement with central hypointense area with gadolinium. A probable diagnosis of pituitary macroadenoma with apoplexy was made and surgery was planned.

**Figure 2.**
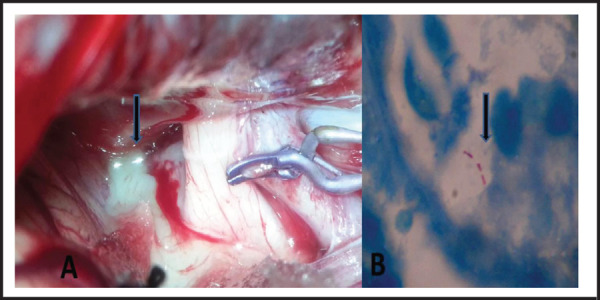
A. Intraoperative picture showing the yellowish contents along (solid arrow) with a clipped ophthalmic aneurysm B. ZN stain showing acid fast bacilli in the cyst contents (solid arrow).

She was given the available options for treatment and largely due to the multiple cranial nerve involvement and large suprasellar extension, she decided and underwent transcranial microscopic pituitary adenectomy. Intraoperatively, an incidental right ophthalmic artery aneurysm was found adherent to the wall of the tumor which was clipped. The tumor had splayed out both the optic nerves which were pale looking and was also adhering to the right oculomotor nerve. On opening the cyst wall, there was flow of thick yellowish pus which was non-foul smelling (Figure 2A).

The cyst wall was lined internally with granulation tissue and was excised partially. There was no evidence of tumor. Microscopic near total removal of the abscess was done with good decompression of bilateral optic and right oculomotor nerve. The pus culture and Gram stain was negative for bacteria. Mantoux test was strongly positive (18 mm). Histopathological examination (HPE) showed giant multinucleated cells and was positive for Zeihl-Neilson stain (ZN) staining (Figure 2B).

Postoperatively, she was started on thyroxine and anti-Tubercular (ATT) chemotherapy with rifampicin (10mg/kg/day), isoniazid (5-10mg/kg/day), pyrazinamide (20-35 mg/kg/day) and ethambutol (15 mg/kg/day) along with Pyridoxine 20 mg/day. Her vision improved in both eyes over few weeks. Humphrey visual field test returned to normal. She completed her 6 months of ATT and is on regular endocrinological, ophthalmological and MRI follow up with no recurrence of the PTA for the past 5 years.

## DISCUSSION

Primary PTA is a rare disease which was first described in 1940.^6^ Tubercular abscess of the brain and tuberculomas of the sellar region are more commonly found than PTA.^7,8^ A preoperative differentiation is very difficult and almost all cases of PTA reported have been diagnosed postoperatively. It is more common in females and due to its rarity, the majority are reported as case report or series.^8^ The most common differential diagnosis is from a pyogenic abscess which can occur secondary to hematogenous, local or through infected cerebrospinal fluid spread.

The common causes of pyogenic abscess are Gram-positive organisms (Staphylococcus spp. and Streptococcus spp.), fungal and Gram negative pathogens (Pseudomonas spp. and Klebsiella spp.). Almost all cases present with signs and symptoms related to the pituitary gland which includes endocrine abnormalities of the anterior pituitary gland, headache, visual abnormalities, diabetes insipidus, cranial nerve palsies or apoplexy.^1–5–8–12^ The hormonal deficiencies may occur in around 30 to 50% of cases and include disorders of growth hormone, gonadotropin and the adreno corticotrophin hormones especially if it involves the hypothalamus. These may be associated with prolactin and thyroid hormone deficiencies. Mantoux test may be falsely positive in endemic areas.^2–4^

MRI is the diagnostic modality and shows a cystic-solid mass in the sellar and suprasellar region isointense on T1 and T2W images with heterogeneous areas and ring enhancement on contrast. Thickening of the pituitary stalk and enhancement of the surrounding dura have also been described.^9,10^ The differential diagnosis of sellar cystic lesions includes carcinoma, adenoma, Rathkes cleft cyst, neurocysticercosis, pyogenic abscess or a craniopharyngioma.^11,12^ Tuberculomas are classically seen in contrast scan as multiple coalescing lesions and the normal pituitary gland is seen separately as a rim. In spite of the various radiological features it is difficult to diagnose PTA with certainty.^2–3–9^

Surgery remains the initial treatment and it is approached through the Trans-sphenoidal, trans-nasal, endoscopic or transcranial route. Stereotactic aspiration, simple decompression of cyst, craniotomy and aspiration or total removal of cyst are the other options available. Intraoperatively it is difficult to differentiate a pyogenic abscess from PTA but can be distinguished from a pituitary adenoma.^13^ Near total removal along with good decompression of the optic nerve/chiasma is important. In our case the cyst was filled with yellowish contents and was adherent to the surrounding dura and optic nerves. An incidental ophthalmic segment aneurysm was also found adherent to the wall which was clipped to avoid complication and surgery in future. Complete decompression including the lateral recess was achieved to free the oculomotor nerve.

Positive Zeihl-Neelsen stain for acid fast bacilli with or without a growth on Lowenstein-Jensen medium at six weeks usually confirms the diagnosis of PTA. Our case had positive ZN stain but a negative culture. All cases of PTA need to complete the ATT course and need long term clinical, radiological and hormonal checkup. Postoperatively there is an acceptable visual/cranial nerve deficit improvement with complete or partial endocrinological improvement. The patients need to be followed serially with hormonal and radiological work-up to diagnose any abnormal findings. There is good clinical improvement postoperatively in this case which emphasizes the importance of early surgical decompression of the PTA.

## Consent

**JNMA Case Report Consent Form** was signed by the patient and the original is attached with the patient's chart.

## Conflict of Interest

**None.**
